# Bisphosphonates in the adjuvant treatment of cancer: experimental evidence and first clinical results

**DOI:** 10.1054/bjoc.1999.1077

**Published:** 2000-03-21

**Authors:** I J Diel, G R Mundy, the International Bone and Cancer Study Group (IBCG)

**Affiliations:** Department of Ob/Gyn, University of Heidelberg, Voss-str 9, Heidelberg, 69115, Germany; University of Texas, Health Science Center, San Antonio, TX, USA

**Keywords:** bone metastases, bisphosphonates, prevention, prophylaxis

## Abstract

Several animal models, as well as a number of cell culture experiments, indicate a prophylactic effect of bisphosphonates in respect of subsequent bone metastasis. Moreover, in preliminary clinical trials involving patients with advanced breast cancer and local or remote metastases, biophosphonates produced a reduction in new skeletal metastases. This overview summarizes and discusses the results of the latest investigations. It opens with a section on the pathophysiology of bone metastasis, which is followed by a report on animal models and first studies of bisphosphonate treatment as a new approach in systemic adjuvant therapy. © 2000 Cancer Research Campaign


British Journal of Cancer (2000) 82(8), 1381–1386
© 2000 Cancer Research Campaign
DOI: 10.1054/ bjoc.1999.1077, available online at http://www.idealibrary.com on IDEAL®

Review

### Bisphosphonates in the adjuvant treatment of cancer: experimental evidence and first clinical results

**IJ Diel[1] and GR Mundy[2] for the International Bone and Cancer Study Group (IBCG)***

[1]Department of Ob/Gyn, University of Heidelberg, Voss-str 9, 69115 Heidelberg, Germany; [2]University of Texas, Health Science Center, San Antonio, TX, USA

**Summary** Several animal models, as well as a number of cell culture experiments, indicate a prophylactic effect of bisphosphonates in respect of subsequent bone metastasis. Moreover, in preliminary clinical trials involving patients with advanced breast cancer and local or remote metastases, biophosphonates produced a reduction in new skeletal metastases. This overview summarizes and discusses the results of the latest investigations. It opens with a section on the pathophysiology of bone metastasis, which is followed by a report on animal models and first studies of bisphosphonate treatment as a new approach in systemic adjuvant therapy. © 2000 Cancer Research Campaign

**Keywords**: bone metastases; bisphosphonates; prevention; prophylaxis

Bisphosphonates are analogues of pyrophosphate and, like pyrophosphate, are strongly bound to hydroxyapatite on the surface of bone. In contrast to pyrophosphate, which is rapidly hydrolysed by phosphatases, bisphosphonates are stable and reduce the number and activity of osteoclasts by various means. This inhibition of bone resorption forms the pharmacological basis for the treatment of tumour-induced osteolysis (Rodan and Fleisch, 1996; Fleisch, 1997). Bisphosphonates provide an osteoprotective effect for the remaining healthy skeleton and can support the remineralization of those bone sections with existing metastatic involvement (Averbuch, 1993; Kanis, 1995).

The ability of bisphosphonates to reduce skeletal morbidity has been shown in numerous clinical trials, particularly in patients with metastatic breast cancer and multiple myeloma. Irrespective of the specific agent or of the route of administration (oral or intravenous), skeletal complications were reduced by some 25–50% (Paterson et al, 1993; Van Holten-Verzantvoort et al, 1993; Berenson et al, 1996, 1998; Hortobagyi et al, 1996, 1998; Bloomfield, 1998; McCloskey et al, 1998). None of the studies, however, was sufficiently powered to demonstrate statistically a benefit in terms of survival of breast cancer patients treated with bisphosphonates except in subgroups.

### PATHOPHYSIOLOGY OF METASTATIC BONE DISEASE

All malignant tumours are potentially capable of metastatic spread to bone, but some have a special predilection to target the skeleton specifically. Breast, prostate, lung, thyroid and renal cell cancers and multiple myeloma all belong to this group, and are jointly responsible for 80–90% of all bone metastases (Weiss and Gilbert, 1981; Galasko, 1986). Apart from the fact that, in the vast majority of cases, metastasis is an indication for incurability, the complications of bone metastases severely affect the quality of life of patients. In breast cancer for example, the commonest complication is bone pain, which affects almost 90% of the patients; some 25% suffer from a pathological fracture or spinal compression syndromes, whereas bone marrow infiltration with suppression of haematopoiesis is observed in 10% of patients. Furthermore, 10–20% of affected women suffer from hypercalcaemic episodes. Tumour osteolysis represents the morphological consequence of these complications (Coleman and Rubens, 1985, 1987; Theriault and Hortobagyi, 1992).

Bone metastases develop according to the same criteria applicable to other metastases, i.e. the tumour releases cells which migrate through the extracellular matrix and penetrate the basement membrane. They are then transported to distant organs via the circulation. In the target organ the process operates in reverse: the metastatic cells enter the perivascular space and are deposited there. This process is mediated partly by adhesion molecules and partly by chemotaxis (Rubens, 1992). Although most of the disseminated cells perish, a few cells are capable of producing micrometastatic proliferation, or remain dormant, only to grow at a later stage. In 30–45% of patients with breast cancer such cells are found in the bone marrow, although it has not yet proved possible to differentiate between those cells that perish and the remainder that remain capable of proliferation (Diel et al, 1994).

Although our understanding of the processes of tumour cell dissemination, cell dormancy and the early division phase is limited, we have nevertheless learned a great deal about the interaction between micrometastases in the bone marrow and the bone and its cell populations. During the early phase of bone metastasis the bone is destroyed not by the tumour itself but rather by the osteoclasts that have been activated by substances secreted in a paracrine and/or autocrine fashion. Of particular importance is parathyroid hormone-related peptide (PTHrP). This peptide is produced by breast cancer cells in the bone metastatic site, and is responsible for osteolysis (Guise et al, 1996). By acting on immune cells or osteoblasts, paracrine osteoclast activation directly or indirectly leads to degradation of the mineralized bone matrix, thereby enabling growth factors and cytokines that were

*Received 24 May 1999*
*Revised 11 October 1999*
*Accepted 4 November 1999*

*Correspondence to*: IJ Diel

*See Appendix.

previously deposited in the bone to accelerate the rate of proliferation of micrometases. The vicious cycle of dialogue between tumour cells and bone cells can be interrupted by the therapeutic use of bisphosphonates, which inhibit osteoclastic bone resorption and thereby decrease production of active growth factors in the bone microenvironment (Mundy, 1991, 1995).

### IN VITRO STUDIES ON THE CYTOTOXICITY OF BISPHOSPHONATES

In the first years of bisphosphonate research only few investigations with cancer cell lines have been able to confirm or refute a direct cytotoxic effect. As early as 1982, however, Reitsma et al were able to demonstrate a cytotoxic effect in macrophages. This effect was achieved with clodronate at therapeutic dosages, and with pamidronate at much higher dosages. Mönkkönen's study group in Finland confirmed this effect of clodronate in numerous investigations, and also discovered that liposome-encapsulated clodronate is many times more potent than the free substance. The same study group also confirmed a cytotoxic effect for etidronate and pamidronate in the liposome-encapsulated form (Mönkkönen et al, 1994). Several studies in recent years have confirmed that the cytotoxic effect in both macrophage-like cells and osteoclasts is achieved by the induction of apoptotic processes (Coxon et al, 1995; Hughes et al, 1995; Rogers et al, 1996). Interestingly, there appear to be differences between the various bisphosphonates. Clodronate induces both necrotic and apoptotic cell death following metabolism to the non-hydrolysable ATP analogue adenosine 5′-(beta, gamma-dichloromethylene)-triphosphate (Selander et al, 1996; Frith et al, 1997). In the case of aminobisphosphonates, apoptosis seems to be caused by inhibition of the mevalonate pathway with subsequent prevention of prenylation (Luckman et al, 1998; Benford et al, 1999; Rogers et al, 1999). There are now several studies in myeloma and breast cancer cell lines that underline this effect for both clodronate and the aminobisphosphonates (Shipman et al, 1997, 1998; Aparico et al, 1998; Busch et al, 1998; Fromigue et al, 1999). It is not yet clear whether this effect merely represents an additional explanation for the mode of action of bisphosphonates or whether the apoptosis of macrophage-like cells and osteoclasts can produce a change in the microenvironment of tumour cells.

Furthermore, two recently published investigations have shown that bisphosphonates change the adhesion properties of tumour cells and the bone surface. Van der Pluijm et al (1996) incubated bone disks with various bisphosphonates and subsequently observed that the disks were resistant to the adhesion properties of tumour cells. The strongest effect was produced by ibandronate and the weakest effect by clodronate. Boissier et al (1997) investigated the adhesion properties of breast and prostate cancer cell lines. After pretreatment with clodronate the adhesiveness of the cells was drastically reduced. There have been no reports to confirm whether this effect of bisphosphonates on adhesion molecules also affects tumour cells that have already disseminated. This question requires investigation in animal models.

### ANIMAL STUDIES

The first evidence confirming a reduction in osteolytic lesions as a result of early bisphosphonate therapy was observed with etidronate (Guaitani et al, 1984; Jung et al, 1984). Using a mouse tumour model and bladder tumour cells, Nemoto et al (1987) showed a reduction in the extent of tumour osteolysis and a prolongation of survival time in treated animals. But the relatively weak effect of etidronate and the impairment of mineralization resulting from its use became apparent at a very early stage in the experiments.

Comprehensive studies on the osteoprotective action of clodronate were conducted by Krempien's study group (Krempien and Manegold, 1993; Krempien, 1994, 1996). In numerous experiments with the PTHrP-producing Walker carcinosarcoma 256, bone was inoculated with tumour cells and the effects subjected to histological investigation. The extent of bone destruction was markedly reduced by pretreatment with bisphosphonates. The degree of destruction correlated with the duration and intensity of the clodronate treatment. The longer the treatment-free interval, the weaker the protective effect on bone. The study group viewed this finding as evidence that a continuous supply of bisphosphonates was more beneficial than interval therapy in terms of prophylaxis.

Krempien and Wingen were also able to show the same osteoprotective effect with pamidronate in the hypercalcaemic Walker tumour. No difference was detected between pamidronate and other bisphosphonates (Wingen et al, 1986; Krempien et al, 1988).

Kostenuik et al (1993) investigated the efficacy of pamidronate in rat bone after the injection of Walker cells. Fisher rats were pretreated for 7 days with pamidronate (0.5 mg $kg^{-1}$) and then given an intramuscular injection of tumour cells. Two weeks later the animals were sacrificed and the bones subjected to histological analysis. Compared to the controls, the cancerous bone volume was three times higher in the animals pretreated with pamidronate. Contrary to expectation, the tumour mass was increased in the pretreated animals without any demonstrable effects on extraosseous metastases.

Contrasting results were reported by Sasaki et al (1995) who observed a reduced tumour burden in bone in nude mice pretreated with the bisphosphonate risedronate and subsequently given an intracardiac injection of cells from a human breast cancer cell line (MDA-MB-231). In a second experiment, risedronate was only administered after the appearance of the first bone metastasis. In both trials, the administration of the bisphosphonate delayed or reduced the occurrence of further skeletal metastases. The pretreated animals also survived for a significantly longer period. Similar findings were observed with the bisphosphonate ibandronate (Yoneda et al, 1997).

In another animal model, Müller et al (1996) inhibited the intraperitoneal growth of myeloma cells by continuous pamidronate injections. In some cases the tumour weight in the test group was reduced by over 50% compared to the controls. Moreover, Hall and Stoica (1994) showed bisphosphonates to be capable of reducing the number, extent and size of bone metastases. In their experiments rats also received an intracardiac injection of a breast cancer cell line (ENU 1564). Thereafter, the animals received the bisphosphonate risedronate, whereas the control animals received physiological saline solution. After 4 weeks the rats were sacrificed and the pattern of metastasis evaluated. Although visceral dissemination was identical in both groups, the animals receiving the adjuvant treatment showed considerably fewer bone metastases ($n$ = 33) than the control group ($n$ = 151). In addition, 30% of the biphosphonate-treated rats were completely free of skeletal metastases, compared to just 16% of the control rats.

© 2000 Cancer Research Campaign

In summary, there is substantial evidence that bisphosphonates can prevent the development of bone metastases in various animal models. These studies justify clinical studies of bisphosphonates as prophylactic agents.

### FIRST CLINICAL TRIALS

At present, the therapy of tumour osteolysis and associated complications is still highly unsatisfactory and is of a purely palliative nature. In this respect it could therefore be very worthwhile to carry out early osteoprotection, at least in patients at a high risk of subsequent skeletal metastasis. Older clinical trials provided initial evidence that patients who were treated with bisphosphonates developed fewer new metastases. In particular, Elomaa et al (1983, 1987) observed this effect after administration of clodronate in a controlled non-randomized study in patients with breast cancer and osseous metastases, although it should be pointed out that the number of patients in each group was small. Following discontinuation of the bisphosphonate, the number of new metastases in the two groups became similar.

### SECONDARY PREVENTION OF METASTASES

The osteoprotective effect of clodronate was first investigated in a double-blind, randomized, placebo-controlled study by Kanis et al (1996). The trial was carried out in patients with advanced breast cancer and local or distant metastases who did not have bone metastases. Sixty-six women received 1600 mg clodronate orally per day for 3 years, whereas 67 women received a placebo for the same period. At the end of treatment it was found that there were fewer new bone metastases in the clodronate group than in the control group (15 vs 19) and that the overall number of metastases was also lower (32 vs 63; $P > 0.005$). As expected, the number of skeletal complications was also lower in the clodronate group.

A non-placebo-controlled, randomized study in patients with breast cancer and already evident bone metastases was published by Conte et al (1996). In this study, 152 patients received chemotherapy and 145 patients received chemotherapy supplemented by infusions of 45 mg pamidronate every 3 weeks. The therapy was continued at least until there was renewed skeletal progression. Although the dosage of pamidronate was low, evaluation of the study revealed prolongation of the skeletal recurrence-free interval (249 vs 168 days; $P = 0.02$) and the complication-free interval (533 vs 490 days); but not a reduction in the number of new metastases. A study published in 1996 by Van Holten-Verzantvoort et al (1996) also failed to show a reduction in the number of metastases. In this study, 142 patients with breast cancer with advanced local or distant disease but without bone metastases were enrolled and were given either continual treatment with 300 mg pamidronate orally or were simply followed up. At the end of the study there were no signs of a reduction in the frequency of metastases. Similar (negative) results were also seen in two trials in 304 myeloma patients and in 610 women with advanced breast cancer but without skeletal metastasis (Ford et al, 1998). Following randomization, the myeloma patients received either 300 mg pamidronate or placebo, whereas the breast cancer patients received either 150 mg pamidronate or placebo (for an unlimited period). Both studies failed to show a reduction in the prevalence of bone metastases. It would, however, be wrong to conclude on this basis that pamidronate is ineffective in the prophylactic setting. Pamidronate is extremely poorly absorbed (< 1%) when given by the oral route. At doses of 600 mg, oral pamidronate is effective in the treatment of tumour osteolysis, but the adverse drug reactions (oesophagitis and gastritis with ulceration) are unacceptable. It is likely that prophylactic studies with intravenous pamidronate might produce better results.

### PRIMARY PROPHYLAXIS OF METASTASES

The first study of the adjuvant use of bisphosphonates in breast cancer was presented in 1997 and has been published since then (Diel et al, 1998). In this trial, which was randomized but not placebo-controlled, 157 patients were treated with 1600 mg clodronate orally per day for 2 years and a further 145 patients served as controls. At the time of primary surgery all patients had immunocytologically detectable tumour cells in bone marrow (minimal residual disease) and were therefore at a high risk of subsequent metastasis (Diel et al, 1996). The study was evaluated after a median follow-up period of 36 months. In the bisphosphonate group there was a significant reduction in both the number of bone metastases ($P = 0.003$), and in the number of non-osseous metastases ($P = 0.003$; overall survival $P < 0.001$). Furthermore, the number of bone metastases per patient was only half as high in the bisphosphonate group as in the control group (3.1 vs 6.3). The authors were surprised by the significant reduction in visceral metastases and suggested that this effect might be due to a cytotoxic effect of chemotherapy and hormone therapy. There is evidence from animal experiments to support this hypothesis (Wingen et al, 1988; Stearns and Wang, 1996).

Some of the results of the Heidelberg study have been confirmed in a report presented at the 1998 ASCO Meeting. In a controlled double-blind study, 1079 women with primary breast cancer received either 1600 mg clodronate or a placebo in addition to standard systemic therapies. In an initial analysis of this Canadian–British–Scandinavian study, there was also a significant reduction in the incidence of bone metastases (Powles et al, 1998). The effect was slightly better in post-menopausal women. With regard to the reduction in visceral metastases, the authors found a trend, but no significant differences. The overall survival time was the same in both groups. At the 1999 ASCO Meeting a third study from Finland was presented that generated completely different results (Saarto et al, 1999). In this trial with 299 patients with node-positive primary breast cancer were also treated with 1600 mg clodronate orally (but for 3 years). The results showed no significant differences with regard to the incidence of bone metastases but indicated a significant increase in visceral metastasis and a deterioration in overall survival.

To this time, no such harmful effects of clodronate have been reported, either in preclinical or clinical trials. Saarto's prevention study is the first of its kind. Should a deleterious effect actually exist, it would have an immense impact, since it might affect other bisphosphonates as well as clodronate. Furthermore, such an effect could not be ignored if the substances are used over a period of years in osteoporosis.

Even so, and neither can this go unmentioned in the analysis of the study Saarto/Elomaa, the best effect was still seen with regard to bone metastases (i.e. not a significant deterioration). Following careful consideration, only methodological differences can explain why the three prophylaxis studies arrived at different results. Only a relatively small number of patients were enrolled (about 300 in each case) in both the Heidelberg and the Finnish studies, neither of which was double-blind. Such small numbers can produce random results. From a methodological point of view the Powles

© 2000 Cancer Research Campaign study is best – it is most trustworthy with its large sample size, and its results lie between those from Heidelberg and those from the Finnish group. The inclusion criteria were different in all three studies. In the largest study, all patients with primary breast cancer were enrolled, compared with node-positive patients in the Finnish study and patients with tumor cells in the bone marrow at the time of surgery in the Heidelberg study. Speculating about micrometastasis as a therapeutic target, this last group of patients could have the best preconditions for prophylaxis, since an apoptotic effect of bisphosphonates accumulated on the bone surface could have an influence on the individual tumour cells.

Because of their contradictory results, the three adjuvant studies indicate the urgent need for new randomized, placebo-controlled studies to confirm or refute the preliminary findings.

### ARGUMENTS FOR THE ADJUVANT USE OF BISPHOSPHONATES

The efficacy and tolerability of most drugs that are presently used in adjuvant systemic therapy of primary malignancies were previously tested in patients in a palliative setting. This is also likely to be the case with the bisphosphonates. There are now numerous studies that demonstrate the effectiveness of the individual bisphosphonates in preventing skeletal complications. The reduction in symptoms by about 25–50% testifies to the efficacy of the bisphosphonates. In comparison to cytotoxic agents, the number and incidence of complications and side-effects are extremely low with bisphosphonates, with levels similar to those reported for tamoxifen. To date, no study has shown any signs of the long-term toxicity on bone that was initially feared with this group of agents. In addition, bisphosphonates are also used for non-oncological indications (e.g. Paget's disease, osteoporosis).

The occurrence of cancer treatment-induced hypogonadism (chemotherapy, endocrine therapy) is an extremely important factor for affected patients, but currently receives too little attention. This may later cause osteoporosis with all the associated complications. Long-term osteoprotection, which can be provided by bisphosphonates, is likely to be helpful in such patients (Saarto et al, 1997).

Animal experiments and initial clinical experience indicate that it is worthwhile to pursue further the question of adjuvant therapy with bisphosphonates. It is not clear whether the major action of the drugs is via a direct cytotoxic effect or via inhibition of growth of micrometastatic cells by changing the microenvironment. However, all investigations to date suggest that it is important to treat the metastatic target organs as well as the primary tumour. The skeleton, with its clear interaction between bone cells and metastatic tumour cells, offers an excellent model for this approach.

It is important to confirm the first but encouraging results of adjuvant bisphosphonate therapies. Candidates for such studies are patients with tumours that metastasize to bone, in particular breast, lung and prostate cancer, and patients with multiple myeloma. In order to obtain beneficial results it would be best to start by recruiting patients at a high risk of metastasis, i.e. oestrogen receptor-positive breast cancer patients with regional lymph node involvement, local progression or presence of tumour cells in bone marrow.

Another method would be to enroll patients with elevated levels of specific prognostic markers for bone metastases. Several studies have shown that these might be patients with primary tumours that produce immunoreactive PTHrP. Patients with PTHrP-positive breast cancers develop bone metastases significantly more frequently than patients with PTHrP-negative tumours (Bundred et al, 1992). Recently it has been suggested that PTHrP positivity may provide a survival advantage (Henderson et al, 1999). A further promising prognostic factor could be the detection of bone sialoprotein (BSP) in the serum of patients with primary breast cancer. In a first study, it has been reported that patients who subsequently developed skeletal metastases had increased serum BSP (Diel et al, 1998). However, because cross-laps (collagen degradation fragments in serum) were elevated in some patients, it is not completely clear whether some of the serum BSP detected is derived from the bone metabolism rather than from the primary tumour (Diel et al, 1999). This could mean that BSP is also an early marker for an onsetting bone metastasis. Possibly, BSP is both a prognostic factor and an early marker, and thus identifies a risk group that would profit from preventive bisphosphonate therapy.

At present it is unclear whether this type of adjuvant therapy with bisphosphonates should be given continually by the oral route, or whether an intravenous interval therapy could produce the same results. It is also uncertain whether the doses used in a palliative setting are optimal or whether lower doses might also suffice. The optimum period of adjuvant treatment is also subject to debate. What is clear, however, is that confirmation of the initial clinical results will open a new chapter in the treatment of malignant tumours.

### REFERENCES

Aparicio A, Gardner A, Tu Y, Savage A, Berenson J and Lichtenstein A (1998) In vitro cytoreductive effects on multiple myeloma cells induced by bisphosphonates. *Leukemia* **12**: 220–229

Averbuch SD (1993) New bisphosphonates in the treatment of bone metastases. *Cancer* **72**: 3443–3452

Benford HL, Frith JC, Auriola S, Mönkkönen J and Rogers MJ (1999) Farnesol and geranylgeraniol prevent activation of caspases by aminobisphosphonates: evidence for two distinct pharmacological classes of bisphosphonate drugs. *Mol Pharmacol* **56**: 131–140

Berenson JR, Lichtenstein A, Porter L, Dimopoulos MA, Bordoni R, George S, Lipton A, Keller A, Ballester O, Kovacs MJ, Blacklock HA, Bell R, Simeone J, Reitsma DJ, Heffernan M, Seaman J and Knight RD (1996) Efficacy of pamidronate in reducing skeletal events in patients with advanced myeloma. *N Engl J Med* **334**: 488–493

Berenson JR, Lichtenstein A, Porter L, Dimopoulos MA, Bordoni R, George S, Lipton A, Keller A, Ballester O, Kovacs MJ, Blacklock HA, Bell R, Simeone J, Reitsma DJ, Heffernan M, Seaman J and Knight RD (1998) Long-term pamidronate treatment of advanced multiple myeloma patients reduces skeletal events. *J Clin Oncol* **16**: 593–602

Bloomfield DJ (1998) Should bisphosphonates be part of the standard therapy of patients with multiple myeloma or bone metastases from other cancers? An evidence-based review. *J Clin Oncol* **16**: 121812–25

Boissier S, Magnetto S, Frappart L, Cuzin B, Ebetino FH, Delmas PD and Clezardin P (1997) Bisphosphonates inhibit prostate and breast carcinoma cell adhesion to unmineralized and mineralized bone extracellular matrix. *Cancer Res* **57**: 3890–3894

Bundred NJ, Walker RA, Ratcliffe WA, Warwick J, Morrison JM and Ratcliffe JG (1992) Parathyroid hormone related protein and skeletal morbidity in breast cancer. *Eur J Cancer* **28**: 690–692

Busch M, Rave-Fränk M, Hille A and Dühmke E (1998) Influence of clodronate on breast cancer cells in vitro. *Eur J Med Res* **3**: 427–431

Coleman RE and Rubens RD (1985) Bone metastases and breast cancer. *Cancer Treat Rev* **12**: 251–270

Coleman RE and Rubens RD (1987) The clinical course of bone metastases in breast cancer. *Br J Cancer* **55**: 61–66

Conte PF, Latreille J, Mauriac L, Calabresi F, Santos R, Campos D (1996) Bonneterre J, Francini G and Ford JM. Delay in progression of bone metastases

© 2000 Cancer Research Campaign in breast cancer patients treated with intravenous pamidronate: results from a multinational randomized controlled trial. *J Clin Oncol* **14**: 2552–2559
Coxon FP, Russell RGG and Rogers MJ (1995) Pathways of bisphosphonate-induced apoptosis in murine macrophage-like cells. *Bone* **17**: 600 (Abstr. 10).
Delmas PD, Balena R, Confravreux E, Hardouin C, Hardy P and Bremond A (1997) Bisphosphonate risedronate prevents bone loss in women with artificial menopause due to chemotherapy of breast cancer: a double-blind, placebo-controlled study. *J Clin Oncol* **15**: 955–962
Diel IJ, Costa SD, Kaufmann M and Bastert G (1994) Detection and characterization of tumor cells in bone marrow of patients with primary breast cancer. In: *Metastatic Bone Disease. Fundamental and Clinical Aspects*, Diel IJ, Kaufmann M and Bastert G (eds), pp. 31–45. Springer: Berlin
Diel IJ, Kaufmann M, Costa SD, Holle R, von Minckwitz G, Solomayer EF, Kaul S and Bastert G (1996) Micrometastatic breast cancer cells in bone marrow at primary surgery: prognostic value in comparison to nodal status. *J Natl Cancer Inst* **88**: 1652–1664
Diel IJ, Solomayer EF, Costa SD, Gollan C, Goerner R, Wallwiener D, Kaufmann M and Bastert G (1998) Reduction in new metastases in breast cancer with adjuvant clodronate treatment. *N Engl J Med* **339**: 357–363
Diel IJ Solomayer EF, Seibel MJ, Pfeilschifter J, Maisenbacher H, Gollan Ch, Pecherstorfer M, Conradi R, Kehr G, Boehm E, Armbruster FP and Bastert G (1999) Serum bone sialoprotein in patients with primary breast cancer is a prognostic marker for subsequent bone metastasis. *Clin Cancer Res* **5** : 3914–3919
Diel IJ, Solomayer EF, Seibel M, Pfeilschifter J, Maisenbacher H, Gollan Ch, Conradi R, Naser W, Hoyle N and Bastert G (1999) Serum bone sialoprotein and crosslaps are both highly predictive for bone metastases in breast cancer. *Proc Am Ass Cancer Res* **40**: Abstr. 2168
Elomaa I, Blomqvist C, Gröhn P, Porkka L, Kairento AL, Selander K, Lambert-Allardt C and Holmström T (1983) Long-term controlled trial with diphosphonate in patients with osteolytic bone metastases. *Lancet* **I**: 146–149
Elomaa I, Blomqvist C, Porkka L, Lambert-Allardt C and Borgström GH (1987) Treatment of skeletal disease in breast cancer: a controlled clodronate trial. *Bone* **8**: 53–56
Fleisch H (1997) *Bisphosphonates in Bone Disease. From the Laboratory to the Patient*, 3rd edn. Parthenon: New York
Ford JM, van Oosterom, Brincker H, Kandra A and Body JJ (1998) Oral pamidronate: negative results from 3 double-blind, placebo-controlled trials in hypercalcemia, myeloma, and the prevention of bone metastases. *Bone* **22**: 3 (abstract B52)
Frith JC, Mönkkönen J, Blackburn GM, Russell RG and Rogers MJ (1997) Clodronate and liposome-encapsulated clodronate are metabolized to a toxic ATP analog, adenosine 5-(beta, gamma-dichlormethylene) triphosphate, by mammalian cells in vitro. *J Bone Miner Res* **12**: 1358–1367
Fromigue D, Siwek B and Body JJ (1999) Bisphosphonates inhibit breast cancer cell proliferation. *Calcif Tissue Int* **64** (abstract P-261)
Galasko CSB (1986) *Skeletal Metastases*. Butterworth: London
Guaitani A, Polentarutti S, Filipeschi S, Marmonti L, Corti F, Italia C, Coccioli G, Donelli MG, Mantovani A and Garattini S (1984) Effects of disodium etidronate in murine tumor models. *Eur J Cancer Clin Oncol* **20**: 685–693
Guise TA, Yin JJ, Taylor SD, Yoneda T, Dallas M, Boyce BF, Kumagai Y and Mundy GR (1996) Evidence for a causal role of parathyroid hormone related protein in the pathogenesis of human breast cancer-mediated osteolysis. *J Clin Invest* **98**: 1544–1549
Hall DG and Stoica G (1994) Effect of the bisphosphonate risedronate on bone metastases in a rat mammary adenocarcinoma model system. *J Bone Miner Res* **9**: 221–230
Henderson MA, Danks JA, Slavin J, Moseley JM, Harris T and Martin TJ (1999) Production of PTHrP by primary breast cancers predicts improved patient survival and decreased bone metastases. *J Bone Miner Res* **14**: (abstract 1082)
Hortobagyi GN, Theriault RL, Porter L, Blayney D, Lipton A, Sinoff C, Wheeler H, Simeone JF, Seaman J, Knight RD, Heffernan M and Reitsma DJ (1996) Efficacy of pamidronate in reducing skeletal complications in patients with breast cancer and lytic bone metastases. *N Engl J Med* **335**: 1785–1791
Hortobagyi GN, Theriault RL, Lipton A, Porter L, Blayney D, Sinoff C, Wheeler H, Simeone JF, Seaman JJ, Knight R, Heffernan M, Mellars K and Reitsma D (1998) Long-term prevention of skeletal complications of metastatic breast cancer with pamidronate. *J Clin Oncol* **16**: 2038–2044
Hughes DE, Wright KR, Uy HL, Sasaki A, Yoneda T, Roodman GD, Mundy GR and Boyce BF (1995) Bisphosphonates promote apoptosis in murine osteoclasts in vitro and in vivo. *J Bone Miner Res* **10**: 1478–1487
Jung A, Bomand J, Mermillod B, Edouard C and Meunier PJ (1984) Inhibition by diphosphonate of bone resorption induced by the Walker tumor of the rat. *Cancer Res* **44**: 3007–3011
Kanis JA (1995) Bone and cancer: pathophysiology and treatment of metastases. *Bone* **17**: 101S–105S
Kanis JA, Powles TJ, Paterson AHG, McCloskey EV and Ashley S (1996) Clodronate decreases the frequency of skeletal metastases in women with breast cancer. *Bone* **19**: 663–667
Kostenuik PJ, Orr FW, Suyama K and Singh G (1993) Increased growth rate and tumor burden of spontaneously metastatic Walker 256 cancer cells in the skeleton of bisphosphonate treated rats. *Cancer Res* **53**: 5472–5477
Krempien B, Wingen F, Eichmann T, Müller M and Schmähl D (1988) Protective effect of a prophylactic treatment with the bisphosphonate 3-amino-1-hydroxypropane-1,1 bisphonic acid on the development of tumor osteopathies in rat: experimental studies with the Walker Carcinosarcoma 256. *Oncology* **45**: 41–46
Krempien B (1994) Morphological findings in bone metastasis, tumorosteopathy and antiosteolytic therapy. In: *Metastatic Bone Disease. Fundamental and Clinical Aspects*, Diel IJ, Kaufmann M and Bastert G (eds), pp. 59–85. Springer: Berlin
Krempien B (1996) Experimental findings on the osteoprotective potential of bisphosphonates against bone metastases and tumor-induced osteopathy: a pleading for an early and preventive administration. In: *Bone Metastasis – Mechanisms and Pathophysiology*, Orr FW and Singh G (eds), pp. 221–244. Landes: Georgetown, TX
Krempien B and Manegold C (1993) Prophylactic treatment of skeletal metastases, tumor-induced osteolysis, and hypercalcemia in rats with the bisphosphonate CL2MBP. *Cancer* **72**: 91–98
Luckman SP, Hughes DE, Coxon FP, Russell RGG and Rogers MJ (1998) Nitrogen-containing bisphosphonates inhibit the mevalonate pathway and prevent posttranslational prenylation of GTP-binding proteins. *J Bone Miner Res* **13**: 581–589
McCloskey EV, MacLennan ICM, Drayson M, Chapman C, Dunn J and Kanis JA (1998) A randomized trial of the effect of clodronate on skeletal morbidity in multiple myeloma. *Br J Haematol* **100**: 317–325
Mönkkönen J, Taskinen M, Auriola SOK and Urtti A (1994) Growth inhibition of macrophage-like and other cell types by liposome-encapsulated, calcium-bound, and free bisphosphonates in vitro. *J Drug Target* **2**: 299–308
Müller M, Green JR and Fabbro D (1996) The bisphosphonate pamidronate inhibits the growth of a murine myeloma cell line in syngeneic mice. *Blood* **88/10**: Abstr. 2333
Mundy GR (1991) Mechanism of osteolytic bone destruction. *Bone* **12**: 1–6
Mundy GR (1995) *Bone Remodeling and Its Disorders*. Dunitz: London
Nemoto R, Uchida K, Tsutsumi M, Koiso K, Sigenori S and Tetsuro S (1987) A model of localized osteolysis induced by the MBT-2 tumor in mice and its responsiveness to etidronate disodium. *J Cancer Res Clin Oncol* **113**: 539–543
Paterson AHG, Powles TJ, Kanis JA, McCloskey EV, Hanson J and Ashley S (1993) Double-blind controlled trial of oral clodronate in patients with bone metastases from breast cancer. *J Clin Oncol* **11**: 59–65
Powles TJ, Paterson AHG, Nevantaus A, Legault S, Pajunen M, Tidy VA, Rosenqvist K, Smith IE, Ottestad L, Ashley S, Walsh G, McCloskey E and Kanis JA (1998) Adjuvant Clodronate reduces the incidence of bone metastases in patients with primary operable breast cancer. *Proc Am Soc Clin Oncol* **17** (Abstract 468)
Reitsma PH, Teitelbaum SL, Bijvoet OLM and Kahn AJ (1982) Differential action of the bisphosphonates (3-amino-1-hydroxypropylidene)-1,1-bisphosphonate (APD) and disodium dichloromethylidene bisphosphonate (CI2MDP) on rat macrophage-mediated bone resorption in vitro. *J Clin Invest* **70**: 927–933
Rodan GA and Fleisch H (1996) Bisphosphonates: mechanisms of action. *J Clin Invest* **97**: 2692–2696
Rogers MJ, Chilton KM, Coxon FP, Lawry J, Smith MO, Suri S and Russel RGG (1996) Bisphosphonates induce apoptosis in mouse macrophage-like cells in vitro by a nitric oxide-independent mechanism. *J Bone Miner Res* **11**: 1482–1491
Rogers MJ, Frith JC, Luckman SP, Coxon FP, Benford HL, Mönkkönen J, Auriola S, Chilton KM and Russell RGG (1999) Molecular mechanism of action of bisphosphonates. *Bone* **24**: 73S–79S
Rubens RD (1992) The nature of metastatic bone disease. In: *Bone Metastases. Diagnosis and Treatment*, Rubens RD and Fogelman I (eds), pp. 1–10. Springer: London
Saarto T, Blomqvist C, Välimäki M, Mäkelä P, Sarna S and Elomaa I (1997*a*) Chemical castration induced by adjuvant cyclophosphamide, methotrexate, and fluorouracil chemotherapy causes rapid bone loss that is reduced by clodronate: a randomized study in premenopausal breast cancer patients. *J Clin Oncol* **15**: 1341–1347
Saarto T, Blomqvist C, Välimäki M, Mäkelä P, Sarna S and Elomaa I (1997*b*) Clodronate improves bone mineral density in postmenopausal breast cancer patients treated with adjuvant antioestrogens. *Br J Cancer* **75**: 602–605

© 2000 Cancer Research Campaign 

Saarto T, Blomqvist C, Virkkunen P and Elomaa I (1999) No reduction of bone metastases with adjuvant clodronate treatment in node-positive breast cancer patients. *Proc Am Soc Clin Oncol* **18** (Abstract 489)
Sasaki A, Boyce BF, Wright KR, Chapman M, Boyce R, Mundy GR and Yoneda T (1995) Bisphosphonate risedronate reduces metastatic human breast cancer burden in nude mice. *Cancer Res* **55**: 3551–3557
Selander KS, Mönkkönen J, Karhukorpi EK, Harkonen P, Hannuniemi R and Vaananen HK (1996) Characteristics of clondronate-induced apoptosis in osteoclasts and macrophages. *Mol Pharmacol* **50**: 1127–1138
Shipman CM, Rogers MJ, Apperley JF, Russell RGG and Croucher P1 (1997) Bisphosphonates induce apoptosis in human myeloma cell lines: a novel anti-tumor activity. *Br J Haematol* **98**: 665–672
Shipman CM, Croucher PI, Russell RGR, Helfrich MA and Rogers MJ (1998) The bisphosphonate incadronate (YM 175) causes apoptosis of human myeloma cells in vitro by inhibiting the mevalonate pathway. *Cancer Res* **58**: 5294–5297
Stearns ME and Wang M (1996) Effects of alendronate and taxol on PC-3 ML cell bone metastases in SCID mice. *Invasion Metastasis* **16**: 116–131
Theriault RL and Hortobagyi GN (1992) Bone metastasis in breast cancer. *Anticancer Drugs* **3**: 455–462
Van der Pluijm G, Vloedgraven H, van Beek E, van der Wee-Pals L, Löwik C and Papapoulos S (1996) Bisphosphonates inhibit the adhesion of breast cancer cells to bone matrices in vitro. *J Clin Invest* **98**: 698–705
Van Holten-Verzantvoort ATM, Kroon HM, Bijvoet OLM, Cleton FJ, Beex LVAM, Blijham G, Hermans J, Neijt JP, Papapoulos SE, Sleeboom HP, Vermey P and Zwinderman AH (1993) Palliative pamidronate treatment in patients with bone metastases from breast cancer. *J Clin Oncol* **11**: 491–498
Van Holten-Verzantvoort ATM, Hermans J, Beex LVAM, Blijham G, Cleton FJ, van Eck-Smit BCF, Sleeboom HG and Papapoulos SE (1996) Does supportive pamidronate treatment prevent or delay the first manifestation of bone metastases in breast cancer patients? *Eur J Cancer* **32**: 450–454
Weiss L and Gilbert AH (1981). *Bone Metastasis*. Hall: Boston
Wingen F, Eichmann T, Manegold C and Krempien B (1986) Effects of new bisphonic acids on tumor-induced bone destruction in the rat. *J Cancer Res Clin Oncol* **111**: 35–41
Wingen F, Pool BL, Klein P, Klenner T and Schmähl D (1988) Anticancer activity of bisphosphonic acids in methylnitrosurea-induced mammary carcinoma of the rat – benefit of combining bisphosphonates with cystatic agents. *Invest New Drugs* **6**: 155–167
Yoneda T, Sasaki A, Dunstan C, Wiliams PJ, Bauss F, DeClerck YA and Mundy GR (1997) Inhibition of osteolytic bone metastasis of breast cancer by combined treatment with bisphosphonate and tissue inhibitor of matrix metalloproteinase-2. *J Clin Invest* **99**: 2509–2517

### APPENDIX

IBCG: R Bartl, Ludwig-Maximilians Universität, Klinikum Grosshadern, Germany; JJ Body, Institut J Bordet, Université Libre de Bruxelles, Belgium; P Burckhardt, CHUV University Hospital, Lausanne, Switzerland; PD Delmas, INSERM Unité 403, Hôpital Edouard Herriot, Université Lyon, France; IJ Diel, Department of Ob/Gyn, University of Heidelberg, Germany; I Elomaa, Department of Radiotherapy and Oncology, University of Helsinki, Finland; D Felsenberg, Osteoporosis Research Group, Klinikum Steglitz, Berlin, Germany; H Fleisch, Effingerstr 40, Berne, Switzerland; JA Kanis, University of Sheffield, Metabolic Bone Unit, Medical School, Sheffield, UK; RA Kyle, Mayo Clinic, Rochester, MN, USA; GR Mundy, University of Texas, Health Science Center, San Antonio, USA; AHG Paterson, Tom Baker Cancer Center, Calgary, Canada; M Pecherstorfer, Wilhelminenspital, Wien, Austria; RD Rubens, Guys Hospital, London, UK. The International Bone and Cancer Study Group is supported in part by a grant from Roche Pharmaceuticals, Basel, Switzerland.

 *© 2000 Cancer Research Campaign*
